# The Use of Metabolomes in Risk Stratification of Heart Failure Patients: Protocol for a Scoping Review

**DOI:** 10.2196/53905

**Published:** 2024-05-23

**Authors:** Umar Gati Adamu, Marheb Badianyama, Dineo Mpanya, Muzi Maseko, Nqoba Tsabedze

**Affiliations:** 1 Division of Cardiology, Department of Internal Medicine School of Clinical Medicine, Faculty of Health Sciences University of the Witwatersrand Johannesburg South Africa; 2 Nutrition and Hypertension Laboratory School of Physiology, Faculty of Health Sciences University of the Witwatersrand Johannesburg South Africa

**Keywords:** metabolomes, metabolomics, heart failure, risk stratification, morbidity, mortality, metabolic abnormality, scoping review protocol, electronic database

## Abstract

**Background:**

Heart failure (HF) is a significant health problem that is often associated with major morbidity and mortality. Metabolic abnormalities occur in HF and may be used to identify individuals at risk of developing the condition. Furthermore, these metabolic changes may play a role in the pathogenesis and progression of HF. Despite this knowledge, the utility of metabolic changes in diagnosis, management, prognosis, and therapy for patients with chronic HF has not been systematically reviewed.

**Objective:**

This scoping review aims to systematically appraise the literature on metabolic changes in patients with HF, describe the role of these changes in pathogenesis, progression, and care, and identify knowledge gaps to inform future research.

**Methods:**

This review will be conducted using a strategy based on previous reports, the *JBI Manual for Evidence Synthesis*, and the Preferred Reporting Items for Systematic Reviews and Meta-Analysis Extension for Scoping Reviews (PRISMA-ScR) guidelines. A comprehensive search of electronic databases (Medline, EBSCOhost, Scopus, and Web of Science) will be conducted using keywords related to HF, myocardial failure, metabolomes, metabonomics, and analytical chemistry techniques. The search will include original peer-reviewed research papers (clinical studies conducted on humans and systematic reviews with or without a meta-analysis) published between January 2010 and September 2023. Studies that include patients with HF younger than 18 years or those not published in English will be excluded. Two authors (UGA and MB) will screen the titles and abstracts independently and perform a full-text screen of the relevant and eligible papers. Relevant data will be extracted and synthesized, and a third author or group will be consulted to resolve discrepancies.

**Results:**

This scoping review will span from January 2010 to September 2023, and the results will be published in a peer-reviewed, open-access journal as a scoping review in 2024. The presentation of the findings will use the PRISMA-ScR flow diagram and descriptive and narrative formats, including tables and graphical displays, to provide a comprehensive overview of the extracted data.

**Conclusions:**

This review aims to collect and analyze the available evidence on metabolic changes in patients with HF, aiming to enhance our current understanding of this topic. Additionally, this review will identify the most commonly used and suitable sample, analytical method, and specific metabolomes to facilitate standardization, reproducibility of results, and application in the diagnosis, treatment, and risk stratification of patients with HF. Finally, it is hoped that this review’s outcomes will inspire further research into the metabolomes of patients with HF in low- and middle-income countries.

**Trial Registration:**

Open Science Framework; https://osf.io/sp6xj

**International Registered Report Identifier (IRRID):**

DERR1-10.2196/53905

## Introduction

Heart failure (HF) is a heterogeneous disease with increasing prevalence and incidence owing to advances in cardiac care and increasing life span [1]. Despite advances in the care of patients with HF, rehospitalization and death rates remain high, especially in those with advanced disease and associated comorbidities [2,3]. The estimated readmission rate at 30 days is 25%, and the 5-year survival rate is 50%, which is as poor as some common cancers [3,4]. Therefore, a knowledge gap remains, and further studies are needed to identify better ways to risk-stratify these patients. HF is a multisystemic disease associated with metabolic abnormalities including inadequate energy (glucose and fatty acids) handling, anabolic-catabolic imbalance, and urea cycle dysfunction [5,6]. Understanding the critical role of metabolic dysfunction in HF has opened a conceptual gap between cardiac metabolism and HF, particularly in the era of precision medicine.

Metabolomics has emerged as a tool for measuring intermediates or metabolomes in various biological samples including plasma, urine, saliva, and tissues [7-9]. Metabolic profiling assesses endogenous and environmental factors, such as dietary intake and physical activity, and integrates genomic, transcriptomic, and proteomic variation. Hence, metabolomes are biomarkers that can be used to identify individuals at risk of developing HF diagnosis and prognostication in patients with HF [10,11]. In a study by Cheng et al [12], metabolomes complemented other cardiac biomarkers in the stratification of patients with acute decompensated HF. Metabolomes correlated better with rehospitalization and all cause death than B-type natriuretic peptide (areas under the curve 0.85 and 0.74, respectively; hazard ratio 3.08; *P*<.001) [12].

Although the use of metabolomes as a stand-alone biomarker or as a component of a multi-marker strategy is challenged partly by the need for more research, scoping reviews may help overcome this by pulling all shreds of evidence into 1 piece. Scoping reviews are used in cases where the literature on a particular topic is limited, and therefore, not harmonized, and they help identify knowledge gaps [13,14]. Further, scoping reviews do not criticize or appraise the existing literature.

The objectives of this scoping review are to systematically appraise the existing literature on the metabolic changes that occur in patients with HF, the platforms for analyzing them, and to describe the knowledge gap to inform future targeted research that will help optimize their usefulness in patients with HF.

## Methods

### Scoping Review Framework

This scoping review will be carried out following the Preferred Reporting Items for Systematic Reviews and Meta-Analysis Extension for Scoping Reviews (PRISMA-ScR) statement [15] and the methods proposed in the updated *JBI Manual for Scoping Reviews* [16,17].

The methodology of this scoping review will include the following stages proposed by the Joanna Briggs Institute (JBI) in developing a protocol and conducting a review, namely (1) identifying the specific research questions, (2) identifying information sources and search strategies, (3) selecting sources of evidence, (4) selecting data items and extracting them, (5) charting the evidence, (6) synthesizing results, and (7) conducting an ethics review and dissemination [16,17]. The PRISMA-ScR method will guide the synthesis and reporting of the existing evidence in the research area in this review. Any subsequent modification will be highlighted in the final publication.

### Research Questions

The scoping review eligibility will be based on the following research questions: (1) What energy sources are available for a healthy heart under normal physiological conditions? (2) What metabolic changes occur in patients with HF? (3) Which analytic modalities are available for metabolomics profiling? (4) What are the roles of metabolomes in the diagnosis, treatment, and risk stratification of patients with HF?

### Identifying the Specific Research Question or Eligibility Criteria

We will adopt the population, concept, and context framework to define the eligibility criteria and direct the search strategy [15].

### Inclusion Criteria

#### Population

The review will include studies in patients with HF from any etiology who are 18 years and older, including ischaemic and nonischaemic causes, either in an inpatient or outpatient setting. We will exclude studies in patients with HF who are younger than 18 years.

#### Concept

The concept of interest is the use of metabolomes in managing patients with HF. The role of metabolomes in patients with HF is being studied and has been found to help diagnose, assess response to therapy, and predict prognosis. Of late, omics technology, including metabolomes, has opened an avenue to explore other biomarkers for HF management. This scoping review aims to assess the metabolomes in individuals with HF and the alterations noted in the systemic circulation, and by extension, the myocardium during the diseased state. Furthermore, the analytical methods used to assay metabolomes will be explored and included. This review will not critique the methods of analysis but describe what was used in estimating various metabolomes. We will exclude papers that include patients younger than 18 years.

#### Context

This scoping review will include papers or studies that describe metabolome use in managing patients with HF. Studies from all parts of the world will be included.

The specific questions that will guide the search, charting, and reporting of the relevant literature and inform the use of the metabolomes include the following: What energy sources are available for a healthy heart under normal physiological conditions? What metabolic changes occur in patients with HF? Which analytic modalities are available for metabolomics profiling? and What are the roles of metabolomes in the diagnosis, treatment, and risk stratification of patients with HF?

### Information Sources and Search Strategies

An experienced medical librarian will be consulted to assist in developing the framework for the search strategy [18] and will follow the 3-step process recommended by the JBI [17]. The search will aim to identify both published and unpublished reviews and studies. The steps are as follows.

First, a preliminary search of PubMed (Medline) will be conducted using the index terms and text words contained in the title and abstract to describe the paper. The initial keywords used included: [Heart Failure OR myocardial failure] AND [metabolomes] AND [metabonomic *] AND [Analytical Chemistry Technique].

Second, all the identified keywords, index terms, Boolean terms (ie, “AND“ ‘AND,“, ’ OR‘ ‘OR, ' ‘OR” NOT,’ ‘NOT'), and a combination of the appropriate medical subject headings terms that describe the papers will be searched in the following databases: PubMed (Medline), EBSCOhost, Scopus, and Web of Science from January 2010 to September 2023 (Textbox 1). We chose January 1, 2010, as the starting date since metabolomic literature became much more robust, and older papers may not be relevant now.

Last, the authors will manually search the reference lists of all relevant sources to obtain additional studies. A gray literature search of conference proceedings, dissertations, and thesis reports will be conducted manually using the study keywords through Google Scholar in the World Health Organization Library and Open Grey. Only studies published in English will be considered for inclusion in this scoping review, as the author’s team is proficient in English.

**Key concepts of the search strategy.**

**Heart failure**

- “heart failure” [MeSH terms] OR Cardiac Failure [text word]) AND (“heart failure” [MeSH terms] OR Heart Decompensation [text word]) AND “heart failure” [MeSH terms] AND “heart failure” [MeSH terms] AND “heart failure” [MeSH terms] AND (“heart failure” [MeSH terms] OR Right-Sided Heart Failure [text word]) AND (“heart failure” [MeSH terms] OR Right Sided Heart Failure [text word]) AND (“heart failure” [MeSH terms] OR Myocardial Failure [text word]) AND (“heart failure” [MeSH terms] OR Congestive Heart Failure [text word]) AND “heart failure” [MeSH terms] AND “heart failure” [MeSH terms] AND “heart failure” [MeSH terms] AND (“heart failure” [MeSH terms] OR Left-Sided Heart Failure [text word]) AND (“heart failure” [MeSH terms] OR Left Sided Heart Failure [text word]

**Metabolomes**

- “metabolome” [MeSH terms] OR Metabolomes [text word]) OR (“metabolome” [MeSH terms] OR Metabolic Profile [text word]) OR (“metabolome” [MeSH terms] OR Metabolic Profiles [text word]) OR Profile [All Fields] OR (“metabolome” [MeSH terms] OR Metabolic Profiles [text word]) OR Metabolic [All Fields]

**Mass spectrometry**

- “mass spectrometry” [MeSH terms] OR Mass spectrometry [text word]) OR (“tandem mass spectrometry” [MeSH terms] OR Tandem mass Spectrometry [text word]) OR (“gas chromatography-mass spectrometry” [MeSH terms] OR Gas Chromatography-Mass Spectrometry [text word]

**Nuclear magnetic resonance spectroscopy**

- “magnetic resonance spectroscopy” [MeSH terms] OR Nuclear Magnetic resonance spectroscopy [text word]

### Selection of Sources of Evidence

The sources of evidence will be developed and selected based on the eligibility criteria that will ensure that specific information relating to the research questions is included in the scoping review (Textbox 2).

The screening of identified studies for the scoping review shall consist of a title and abstract screening to identify eligible publications and full-text screening to choose papers based on the study’s inclusion and exclusion criteria. The search results will be exported into EndNote version 20, and the reference management system and duplicates will be removed. The authors (UGA and MB) will independently search the title and abstract of all studies for assessment against the inclusion criteria for the scoping review. The potentially relevant sources will be fully retrieved including their citation details. We (UGA and MB) will critically assess the full text of the selected citations against the inclusion criteria. The team will note the reasons for excluding full texts and report in the scoping review. Discrepancies in the study selection will be resolved by a team member (DM, MM, or NT) or by consensus. The authors will undergo in-house training on how to extract before the complete screening of relevant full-text papers. Intra and interobserver reliability will be assessed using Cohen κ coefficient (X) statistics [19].

**Inclusion and exclusion criteria.**

**Inclusion criteria**

- Study design: all study types (observational, cross-sectional, retrospective, prospective, quantitative, and qualitative designs)
- Publication: original, peer-reviewed publications between January 1, 2010, and September 30, 2023, in English
- Study location: all health care settings, no location restrictions
- Study participants: patients with heart failure (HF) 18 years and older
- Studies of cohorts of patients with HF using metabolomes with emphasis on pathophysiology, diagnosis, treatment, and follow-up

**Exclusion criteria**

- Papers like case series and conference abstracts
- Papers that assessed metabolomes without addressing their usefulness in the diagnosis and follow-up of patients with HF
- Papers that included patients with HF younger than 18 years

### Data Extraction

Two authors (UGA and MB) will independently extract data that fit the inclusion criteria from the included studies in the scoping review using the Microsoft Excel form developed by the reviewers (Textbox 3). The data to be extracted includes the paper characteristics (first author, journal, year of publication, study design, study objectives [aims], country of origin); the population characteristics (number of participants, mean age or median with IQR, percentage of males, and diagnosis); the concepts (samples, analytical methods, metabolomes, primary findings, and comments); and the contexts (inpatient or outpatient, globally).

The Microsoft Excel form shall be reviewed, pretested, and modified as necessary by the authors during the data extraction in an iterative process. The reasons for change will be outlined and presented as an appendix for the review. Any discrepancies will be resolved through discussion or by any of these reviewers (DM, MM, and NT).

**Extraction instrument depicting the details of the included studies.**

**Paper characteristics or context**

- First author, journal, year of publication, and country of origin
- Study design
- Study aims
- Inpatient or outpatient

**Population characteristics**

- Population, N
- Mean age, median with IQR, percentage male

**Concept**

- Sample type
- Analytical method
- Metabolomes assessed
- Summary of findings

### Charting the Evidence

The authors will present the characteristics of each of the included studies in a tabular form. We will compare the charted data to ensure uniformity and inconsistencies resolved by arbitration. If additional categories emerge during data extraction, they will be noted and discussed during meetings, and the charting will be amended accordingly. The authors will not perform the bias assessment because it is a scoping review. This modality is in keeping with the recommendations of the PRISMA-ScR [3,15].

### Synthesis of Results

The details of the study selection process will be shown using a flowchart (PRISMA-ScR) of the databases searched, included, and excluded through the steps of a scoping review [18]. For this scoping review, the potential eligible papers will be counted, charted, synthesized, and summarized using descriptive statistics. In some cases, figures and images will be provided to enhance clarity. Since scoping reviews aggregate and summarize available evidence, there will be no critical appraisal of the quality of included papers. The patient and the public will not be involved in developing the protocol.

### Ethics Approval

This scoping review protocol is part of a research work and has been approved by the Human Research Ethics Committee (Medical) of the University of the Witwatersrand, Johannesburg (M220519).

## Results

The results from the scoping review will be presented in a narrative form. The PRISMA-ScR flow diagram will be used to depict the study selection process, the number of papers initially identified and included, and the reason for exclusion. In addition, the charted details, including the title, year of publication, country, study design, characteristics of study participants, study objectives, sample, and analytical methods, will be presented in diagrams or tabular format. The result of this scoping review will be disseminated at conferences, and the paper will be published toward the end of the second quarter of 2024.

## Discussion

### Principal Findings

The discussion section will address various questions that inform the scoping review process. One such is how the heart uses various substrates in health and disease to generate energy in the form of adenosine triphosphate and the various analytical methods used to analyze metabolomes. We will discuss how the heart switches from using the preferred fatty acids to other substrates. We will itemize the papers studied on each substrate during the study period. Analyzing substrate used by the heart in health and disease and finding pathways commonly affected in HF phenotypes will inform future research directions. Furthermore, we will explore the patients with HF studied in the included studies, discuss the knowledge gaps, and the implications for future research efforts. The scoping review will synthesize the evidence from the included papers on the usefulness of metabolomes in the diagnosis, treatment, risk-stratification, and prognosis of patients with HF, with particular attention to the assumptions and limitations. We will, however, not undertake the formal risk of bias evaluation. This information may provide valuable insights into the current state of the literature and highlight areas where improvements may be needed and hope to stimulate further research into metabolomes of HF patients in low- and middle-income countries.

### Limitations

A potential limitation of this scoping review could be the inability to access the full text of some of the included papers; hence, the contents of such references cannot be synthesized. However, efforts will be made to contact the authors of these papers to get those texts or elements relevant to the review. The strength of this scoping review is its ability to examine the state of science and subsequently identify knowledge gaps and directions for future research.

### Conclusions

The prevalence of HF is increasing despite the continued development of several diagnostic and treatment modalities; it is, therefore, imperative to search for biomarkers that will better characterize patients with HF and ensure targeted therapy. The European Society of Cardiology guidelines for the diagnosis and treatment of acute and chronic HF implored clinicians to search for other more specific biomarkers, and hence this scoping review will help synthesize available literature on the use of metabolomes in patients with HF, identify the knowledge gaps, and recommend future research.

## Abbreviations

HF: heart failure
JBI: Joanna Briggs Institute
PRISMA-ScR: Preferred Reporting Items for Systematic Reviews and Meta-Analysis Extension for Scoping Reviews

## Data Availability

The data sets generated or analyzed during this study (protocol) and the scoping review are available from the corresponding author on reasonable request.

## References

[1] Roger VL (2021). Epidemiology of heart failure: a contemporary perspective. Circ Res.

[2] Conrad N, Judge A, Canoy D, Tran J, Pinho-Gomes AC, Millett ERC, Salimi-Khorshidi G, Cleland JG, McMurray JJV, Rahimi K (2019). Temporal trends and patterns in mortality after incident heart failure: a longitudinal analysis of 86 000 individuals. JAMA Cardiol.

[3] Emmons-Bell S, Johnson C, Roth G (2022). Prevalence, incidence and survival of heart failure: a systematic review. Heart.

[4] Tsao CW, Lyass A, Enserro D, Larson MG, Ho JE, Kizer JR, Gottdiener JS, Psaty BM, Vasan RS (2018). Temporal trends in the incidence of and mortality associated with heart failure with preserved and reduced ejection fraction. JACC Heart Fail.

[5] Hunter WG, Kelly JP, McGarrah RW, Kraus WE, Shah SH (2016). Metabolic dysfunction in heart failure: diagnostic, prognostic, and pathophysiologic insights from metabolomic profiling. Curr Heart Fail Rep.

[6] Wende AR, Brahma MK, McGinnis GR, Young ME (2017). Metabolic origins of heart failure. JACC Basic Transl Sci.

[7] Lanfear DE, Gibbs JJ, Li J, She R, Petucci C, Culver JA, Tang WHW, Pinto YM, Williams LK, Sabbah HN, Gardell SJ (2017). Targeted metabolomic profiling of plasma and survival in heart failure patients. JACC Heart Fail.

[8] Wang CH, Cheng ML, Liu MH, Fu TC (2019). Amino acid-based metabolic profile provides functional assessment and prognostic value for heart failure outpatients. Dis Markers.

[9] Bayes-Genis A, Liu PP, Lanfear DE, de Boer RA, González A, Thum T, Emdin M, Januzzi JL (2020). Omics phenotyping in heart failure: the next frontier. Eur Heart J.

[10] McGarrah RW, Crown SB, Zhang GF, Shah SH, Newgard CB (2018). Cardiovascular metabolomics. Circ Res.

[11] Tahir UA, Katz DH, Zhao T, Ngo D, Cruz DE, Robbins JM, Chen ZZ, Peterson B, Benson MD, Shi X, Dailey L, Andersson C, Vasan RS, Gao Y, Shen C, Correa A, Hall ME, Wang TJ, Clish CB, Wilson JG, Gerszten RE (2021). Metabolomic profiles and heart failure risk in black adults: insights from the Jackson Heart Study. Circ Heart Fail.

[12] Cheng ML, Wang CH, Shiao MS, Liu MH, Huang YY, Huang CY, Mao CT, Lin JF, Ho HY, Yang NI (2015). Metabolic disturbances identified in plasma are associated with outcomes in patients with heart failure: diagnostic and prognostic value of metabolomics. J Am Coll Cardiol.

[13] Levac D, Colquhoun H, O'Brien KK (2010). Scoping studies: advancing the methodology. Implement Sci.

[14] Munn Z, Peters MDJ, Stern C, Tufanaru C, McArthur A, Aromataris E (2018). Systematic review or scoping review? guidance for authors when choosing between a systematic or scoping review approach. BMC Med Res Methodol.

[15] Tricco AC, Lillie E, Zarin W, O'Brien KK, Colquhoun H, Levac D, Moher D, Peters MDJ, Horsley T, Weeks L, Hempel S, Akl EA, Chang C, McGowan J, Stewart L, Hartling L, Aldcroft A, Wilson MG, Garritty C, Lewin S, Godfrey CM, Macdonald MT, Langlois EV, Soares-Weiser K, Moriarty J, Clifford T, Tunçalp Ö, Straus SE (2018). PRISMA extension for Scoping Reviews (PRISMA-ScR): checklist and explanation. Ann Intern Med.

[16] Arksey H, O'Malley L (2005). Scoping studies: towards a methodological framework. Int J Soc Res Methodol.

[17] Peters MDJ, Marnie C, Tricco AC, Pollock D, Munn Z, Alexander L, McInerney P, Godfrey CM, Khalil H (2020). Updated methodological guidance for the conduct of scoping reviews. JBI Evid Synth.

[18] Bramer WM, de Jonge GB, Rethlefsen ML, Mast F, Kleijnen J (2018). A systematic approach to searching: an efficient and complete method to develop literature searches. J Med Libr Assoc.

[19] McHugh ML (2012). Interrater reliability: the kappa statistic. Biochem Med (Zagreb).

